# Stitched textile-based microfluidics for wearable devices†

**DOI:** 10.1039/d4lc00697f

**Published:** 2024-11-18

**Authors:** Martin Hanze, Andrew Piper, Mahiar Max Hamedi

**Affiliations:** a Department of Fibre and Polymer Technology, School of Engineering Sciences in Chemistry, Biotechnology and Health, KTH Royal Institute of Technology Teknikringen 56-58 SE-100 44 Stockholm Sweden mahiar@kth.se; b Institut Català de Nanociència i Nanotecnologia (ICN2), CSIC and The Barcelona Institute of Science and Technology (BIST) Campus UAB Bellaterra 08193 Barcelona Spain andrew.piper@icn2.cat

## Abstract

Thread-based microfluidics, which rely on capillary forces in threads for liquid flow, are a promising alternative to conventional microfluidics, as they can be easily integrated into wearable textile-based biosensors. We present here advanced textile-based microfluidic devices fabricated by machine stitching, using only commercially available textiles. We stitch a polyester “Coolmax®” yarn with enhanced wicking abilities into both hydrophobic fabric and hydrophobically treated stretchable fabric, that serve as non-wicking substrates. In doing so we construct textile microfluidics capable of performing a wide variety of functions, including mixing and separation in 2D and 3D configurations. Furthermore, we integrate a stitched microfluidic device into a wearable T-shirt and show that this device can collect, transport, and detect sweat from the wearer's skin. These can also be machine-washed, making them inherently reusable. Finally, we integrate electrochemical sensors into the textile-based microfluidic devices using stitched gold-coated yarns to detect analytes in the microfluidic yarns. Our stitched textile-based microfluidic devices hold promise for wearable diagnostic applications. This novel, bottom-up fabrication using machine stitching is scalable, reproducible, low-cost, and compatible with the existing textile manufacturing industry.

## Abstract 2

Advanced textile-based microfluidic devices were fabricated by machine stitching, using only commercially available textiles and yarns. These devices perform advanced 3D microfluidics, and electrochemical sensing using stitched gold-coated yarns.

## Graphical abstract

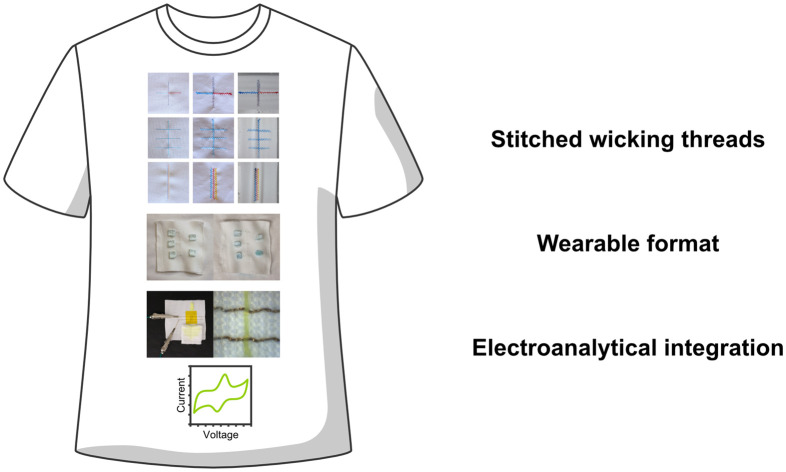

**Tribute to George Whitesides**

In our first meeting in 2013, George asked if I knew about the story. He was talking about the stories told in research articles, because George deeply understands that communication and storytelling are integral parts of the scientific process. He spent countless hours working on my articles, as he did with all his students. I even delivered printed drafts or “outlines” to his house some weekends. If he was lucky, he would also get an apple pie so he wouldn't run out of energy.

George has a unique personality and mind; with deep knowledge across a wide range of scientific topics he could move seamlessly between the finest details and the big picture. I saw him ask a simple but profound question to a Nobel laureate, which could not be answered, and I saw him dig deep into a simple engineering detail because he knew that solving this practical problem could be as crucial as addressing curiosity-driven basic scientific questions. This is his holistic view of the scientific process.

Only a hard-working person with such a unique mind can pioneer dozens of scientific fields, publish over a thousand high-quality articles, and translate science into several billion-dollar ventures. His main achievement, however, is the mentoring of hundreds of successful researchers, many of whom are now connected through powerful bonds and contribute to the global scientific endeavor. This fact is also apparent from the range of interesting and novel topics covered in this special collection. Happy birthday, George!

*Max Hamedi*

## Introduction

The field of microfluidics has opened new ways to manipulate and analyze fluids on the microscale. A promising application area for microfluidics is point-of-care diagnostics and other point-of-need analytical testing, *e.g.*, veterinary testing,^1–3^ food safety,^4,5^ and environmental monitoring.^6–8^ Conventional microfluidics based on silicon, glass, or polymers like polydimethylsiloxane, are widely used in advanced laboratory settings but typically rely on sophisticated microfabrication processes.^9–12^ These technologies have achieved some commercial successes, for example Standard Biotools Inc. where they are used in high-end products, like biochips, however, these rely on auxiliary pumping equipment.^13,14^ Conventional microfluidics have underperformed in producing low-cost products at scale due to the need to incorporate pumps, vacuums and/or power sources. Instead, paper-based microfluidics have been proposed for the next generation of low-cost scalable microfluidics, especially toward advanced paper-based molecular diagnostic devices.^15–18^ However, one application that cannot be met by paper microfluidics is that of wearable diagnostics. Even though wearable diagnostic device research is rapidly increasing, with many examples of advanced devices for sweat sensing, and more, being reported, these devices predominantly rely on conventional microfluidic platforms that are not inherently suited to wearable applications since they are rigid and not breathable, often requiring adhesives to stick them to the wearer.^19–24^ Textile-based microfluidics are a promising alternative;^25–34^ like paper-based microfluidics, the liquid flow in textile-based microfluidics is driven by capillary forces from the microscale channels between individual fibers or fibrils.^35,36^ Threads and fabrics can be made from various affordable materials, including natural fibers (*e.g.* cotton) and synthetic polymers (*e.g.* polyester or nylon). Textile-based microfluidic devices have the potential to be truly wearable and can be seamlessly integrated into the clothing that we wear.^20–24^

The simplest form of thread-based devices uses a single suspended thread in which liquid flows from one end to the other, for colorimetric detection.^37–44^ Conceptually these can therefore be thought of as alternatives to nitrocellulose in conventional lateral flow assays. The need to suspend the threads puts limitations on their integration into more complex analytical devices incorporating advanced microfluidics and electroanalytic capabilities.

“Bottom-up” approaches of integrating advanced microfluidic functionality into wearable devices have been reported by weaving or knitting functional threads into textile fabrics.^45^ This approach, however, requires advanced textile fabrication machines to be scaled up and also limits the choice of threads that can be used, as these have to be compatible with the industrial knitting or weaving machines. Alternatively, a “top-down” approach, in which functional threads are stitched into fabrics is an option. However, most publications on the topic of stitched thread-based microfluidics,^46–54^ stitched electroanalytical devices,^55–58^ and stitched electronics^59–64^ use hand-stitching. Hand stitching is time-consuming and prone to human handling errors. When sewing machines are utilized, the user can control the tension, patterns, spacings, and stitch type with high precision, reproducibility, and accuracy on a large scale. To achieve true compatibility with the common practices and workflow of the textile industry, to be truly scalable, conventional sewing machines need to be used.

Herein we present the first demonstration of the fabrication of advanced machine-stitched textile microfluidic devices. Our work relies on the use of commercially available threads and fabrics. More specifically we show that a commercially available Coolmax® thread, originally designed for superior wicking, can be stitched onto hydrophobic textiles to create advanced three-dimensional textile microfluidic structures. Furthermore, we stitch gold coated threads in combination with the fluidic threads to realize advanced microfluidic and electroanalytical devices. This work opens the path to realize the next generation of advanced fully textile-based micro total analytical devices that are compatible with large scale textile manufacturing.

## Experimental

### Materials

The gold-coated multifilament threads were purchased from Swicofil AG (Emmenbrücke, Switzerland), the nylon thread was purchased from Selfmade Sverige AB (Malmö, Sweden) and the Coolmax® thread sample was donated by Dúctel, s.a. (Barcelona, Spain) (see Table S1, ESI,† for the specification of all the yarns and threads). Teflon-coated woven fabric and stretchable knitted fabric were purchased from tyg.se (fabfab GmbH, Schenefeld, Germany) (see Table S2, ESI,† for the specification of all fabrics). Ferricyanide (FCN) and potassium chloride were purchased from Sigma Aldrich (Sweden). Red, yellow, blue, and green food coloring dyes (carmine, carotene, brilliant blue, and a mix of carotene and brilliant blue, respectively) were produced by Dr. Oetker and purchased from a supermarket in Sweden. Non-woven cotton pads were purchased from a pharmacy in Sweden. Imprenex Wash In (Herdins Färgverk AB, Falun, Sweden) water-repellant agent was bought from Tvättgiganten Sverige AB (Arlandastad, Sweden).

### Scanning electron microscopy

Characterization of the structures, surface, and cross-sections of the Coolmax® Eco yarn was performed by first coating with chromium ions with a 208HR Sputter Coater (Cressington, Watford, UK) and then visualizing with a Hitachi S-4800 Scanning Electron Microscope (Hitachi, Tokyo, Japan) with BSE detector.

### Water-repellant treatment of flexible textiles

Adapting the manufacturer's instructions, the knitted, flexible textile was soaked in 40 °C Milli-Q water with 5.6% (v/v) Imprenex Wash In solution (three times the minimum recommended amount) for 20 min. The textiles were stirred by hand and squeezed every 5 minutes. Afterward, the strips were rinsed with room temperature Milli-Q and then put in an oven at 75 °C until dry (*circa* 1.5–2 hours). The change in hydrophobicity was verified by contact angle measurements using a Goniometer (Ossila B.V., Leiden, Netherlands) and the data analyzed with ImageJ (National Institutes of Health, Bethesda, MD, USA) with the Contact Angle plug-in (Contact_Angle.jar) by Marco Brugnara (available at https://imagej.net/ij/plugins/contact-angle.html at the time of writing). Comparison between data groups was analyzed with unpaired, two-tailed Student's *t*-test in Prism 10 (GraphPad, Boston, MA, USA) with a significance level *α* = 0.05.

### Winding yarn onto spools and bobbins

The Coolmax® was supplied on a large conical spool meant for knitting machines. To transfer the thread to a smaller spool compatible with the sewing machine, the smaller spool was glued on top of a bobbin and the bobbin side was attached to the sewing machine's bobbin winder shaft. The spool was wound by using the bobbin winding function with the thread only being threaded around the thread guide and under the pretension disk. The Coolmax® was distributed evenly over the spool by manually alternating the height of the thread while winding. All threads that were used were transferred from their respective spools to bobbins using the bobbin winder function according to the manufacturer's instructions.

### Fabrication of microfluidic test platforms

All microfluidic test platforms were stitched using a Brother Innovis F480 (Brother, Nagoya, Japan) sewing machine. Straight interlock stitches (program mode 1-02) were used for the woven fabric with a stitch target length of 2.5 mm. Zigzag interlock stitches (program mode 1-10) were used for the knitted fabric with a target width of 3 mm and a target length of 2 mm. The tension was set at 4.0 units for Coolmax® and nylon threads and 5.0 units for the gold-coated threads. Both the Coolmax® and the gold-coated threads were used separately as both spool thread and bobbin thread unless otherwise noted.

Three-dimensional test platforms were fabricated by stitching patterns with straight interlock stitches onto two pieces of woven fabric with Coolmax® as the spool thread and non-wicking nylon thread as the bobbin thread. Where gaps were needed, stitches were cut and unpicked. The two pieces of fabric were stitched together with the side of the nylon threads facing each other. Vertical channels between the two layers were made by stitching, with Coolmax® thread as both bobbin and spool thread, in place back and forth three times, ensuring that the vertical channels touched both stitched patterns on the fabrics for an uninterrupted flow channel.

Devices that stored chemical compounds were made as follows: two collinearly aligned regions of Coolmax® were stitched into the woven fabric with a zigzag pattern (program mode 1-10, 2.5 mm target width, 1.6 mm target length, tension 4.0 units) with a length of about 1 cm. Undiluted food dye was added to the stitches and allowed to dry under ambient conditions. Afterward, a microfluidic channel made with a regular straight stitch was stitched over the zigzag stitch. The device was tested by adding Milli-Q water to one end of the microfluidic channel.

Devices for electroanalytical detection were fabricated as follows: two straight stitches of gold plasma-coated yarn were first stitched in the woven fabric to act as electrodes. Four or twelve rectangular pieces of non-woven cotton (cut from circular cotton pads) roughly 3 × 5 mm in size were stitched onto the fabric parallel to and at an equal distance (around 7 mm) from the electrodes with Coolmax® yarn. The stitches were extended to two full stitches beyond the electrode stitches, ensuring adequate overlap of the fluidic and conductive threads. The sensing region was defined by masking with 3 mm wide tape strips covering both electrodes as well as the in-between and neighboring parts of the microfluidic channel and then drop-casting with a solvated polymer (nail varnish) on either side of the mask. Finally, the stitched crosses were covered with pieces of heat-resistant tape as protection against evaporation. See Fig. S5† for photographs showing the fabrication process step-by-step.

Absorption pads for continuous flow devices were made by cutting nonwoven cotton pads into rectangles of about 2 × 3 cm. Five rows of straight Coolmax® stitches were sewn in parallel and evenly distanced on the cotton pad along the long axis. The continuous devices were constructed the same way as the other electrochemical devices, only with a single stitched microfluidic channel ending in a stitched-in absorption pad.

### Wicking behavior

The wicking behavior of aqueous solutions in the microfluidic channels was tested in both the woven and knitted fabric. Five stitched microfluidic channels (straight or zig-zag) of a length of approximately 13 cm were stitched to the corresponding fabric (woven and knitted, respectively). 10 μL food dye (diluted 1 : 3 with Milli-Q water) was added to one end of each microfluidic channel. The flow was recorded against a ruler with an EOS Rebel T5i (Canon, Tokyo, Japan) and was studied by collecting data points for the wicking distance every 10 s for 120 s. The behavior in the knitted fabric was tested in both stretched and native states.

The comparison of the flow distance increased and twisted fabric was performed as follows: three stitched microfluidic channels (straight or zig-zag) of a length of approximately 12 cm were stitched to the corresponding fabric (woven and knitted, respectively). The fabric devices were either twisted 360 degrees, compressed until three creases were formed (measures were made to not allow the microfluidic channels on either side of the creases to touch), or left in their native state. 30 μL food dye (diluted 1 : 3 with Milli-Q water) was added to one end of each microfluidic channel and the flow was allowed to come to a complete stop before the flow distance was measured.

### Washing textile devices

The effect of washing the textiles was determined by stitching three microfluidic channels in the woven and knitted textiles. These textile devices were then stitched with nylon thread onto a cotton elastane mix T-shirt with an intermediate layer of each respective fabric to prevent the T-shirt's fabric from absorbing liquid from the microfluidic channels. The 15 μL food dye solution was added to each channel and the flow was recorded against a ruler. The food dye was allowed to dry into the stitched microfluidic channel.

The T-shirt with the stitched textile devices was washed in a commercial washing machine (model number EWW148540W, Electrolux, Stockholm, Sweden) with 25 mL liquid detergent (Neutral Cares for sensitive skin & environment Color, Neutral, Copenhagen, Denmark) with the Wool/Handwash setting (30 °C, 1000 rpm centrifugation), in accordance with the cleaning instructions of both fabrics. The detergent is specified to contain 15–30% non-ionic surfactants, 5–15% anionic surfactants, a total of <5% enzymes and phosphonates, as well as several other compounds. After washing, the wicking length was tested again in each stitched microfluidic channel as before, for a total of three rounds of washing.

### Sweat detection in a worn microfluidic device

Moisture indicators were made by fully soaking 1 × 1 cm cotton pads in 1.2 M copper sulfate (aq). The cotton pads were allowed to dry and then heated in an oven at 120 °C for 20 minutes to dry the copper sulfate. Five moisture indicator pads were stitched into the woven fabric. Three were connected with a branched stitched microfluidic channel, and two that were separated from the microfluidic channels were left as positive and negative controls. The textile device was then stitched with nylon thread onto a cotton elastane mix T-shirt with an intermediate layer of fabric to prevent the T-shirt's fabric from absorbing liquid from the microfluidic channels. A small cotton pad acting as a sample collection pad was stitched in place within the T-shirt through all three layers of fabric, connecting the inner side of the T-shirt with the microfluidic channel on the top face of the fabric. Sweating was induced by vigorous exercise for one hour and the results were documented afterwards. The positive control was fully soaked in artificial sweat while the negative was left dry.

### Cyclic voltammetry

CVs of ferricyanide solutions on microfluidic platforms with stitched electrodes of gold-coated threads were obtained with an Autolab PGSTAT204N with MUX 16 module (Metrohm Autolab, Sweden) and the accompanying NOVA 2.1.4 software package. The voltage window used was −0.35 V to 0.35 V and the scan rate was 10 mV s^−1^, unless otherwise stated.

### Chronoamperometry

Chronoamperometric measurements were performed on the stitched electroanalytical devices allowing continuous flows using an Autolab PGSTAT204N with MUX 16 module (Metrohm Autolab, Bromma, Sweden) with the accompanying NOVA 2.1.4 software. Before the start of the measurements, 5 mM ferricyanide solution was allowed to flow through the devices until the microfluidic channels, including the branching stitches in the absorption pads, were visibly fully wetted (roughly 15 minutes with 20 μL additions every 3 minutes). Chronoamperometry was performed by applying a reducing potential of −0.15 V for 24 minutes. At every 3 minute mark, 20 μL ferricyanide solutions of increasingly higher concentrations were added to the sample inlet pad, starting at 5 mM and increasing by increments of 5 mM for every addition up to 40 mM. Data points were collected every 0.5 seconds. The data was normalized against the drift, by subtracting the contribution of the drift by averaging the slope in all the plateau regions across all three devices, calculated in Excel 2019 (Microsoft, Redmond, WA, USA). The data was also smoothed with a 30-point window with a second-order polynomial function with Origin 2020 9.7.0.188 (OriginLab Corporation, Northampton, MA, USA).

## Results and discussion

### Choice of threads and textiles

**Fig. 1:**
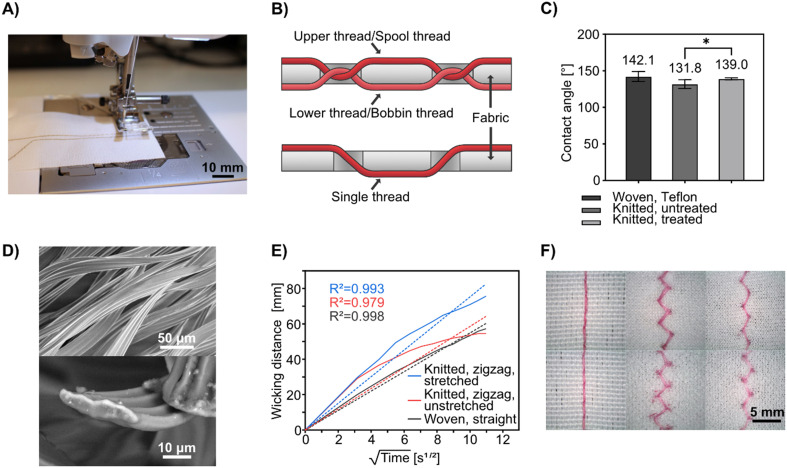
Characterization of fabrics and stitched Coolmax® threads. A) Photograph showing the fabrication process of stitched textile-based microfluidic devices with a conventional sewing machine. Here the spool and bobbin threads were both Coolmax® and the machine was prepped to stitch a microfluidic channel across a pair of pre-stitched gold thread electrodes. B) Schematic illustration of a basic lockstitch compared to a hand-sewn stitch. C) Contact angle measurements on the woven fabric and the knitted fabric. The values are averages of three 9 μL drops with two replicate measurements on each drop (*n* = 6). Legend: asterisk (*) means significant difference (*p* ≤ 0.05). D) SEM pictures of the Coolmax® thread. Top: Side-view. Bottom: Cross-section. E) Wicking behavior of the Coolmax® thread when stitched in the woven and knitted fabrics. Wicking distance is plotted against the square root of time and fitted to the Washburn equation. F) Magnifications of the sewn stitches in the fabrics. From left to right: Straight interlock stitches in woven fabric, zigzag interlock stitches in knitted fabric (unstretched), zigzag interlock stitches in knitted fabric (stretched). The top row shows the upper thread on the right sides of the fabrics and the bottom row shows the bobbin thread on the wrong sides. The fabrics are oriented with the weft and wale directions horizontally, respectively. Captured with a Dino-Lite RS Pro (AnMo Electronics Corporation, Taiwan).

Coolmax® is the brand name for polyester yarns that have superior wicking properties; they are typically used in fabrics designed for sports clothing. This makes the garment “breathe” as the yarn transports sweat throughout the fabric, making it evaporate more easily. Its wicking properties come from its scalloped-oval cross-section which increases the surface area and creates capillary channels along the length of the yarn (see Fig. 1D for SEM images of the yarn cross-section). We have previously used Coolmax® in hand-woven textile-based microfluidic devices.^65,66^ However, those yarn variants were unsuitable for stitching and tended to break in conventional sewing machines. In this project, we have switched to a stitchable Coolmax® thread (Table S1†).

To enable stitched microfluidic structures, we rely on hydrophobic fabrics as the base substrate, so that liquids are constrained to the stitched threads and do not wick and spread to the fabric. Both stretchable and non-stretchable fabrics were used in this project. As a non-wicking, non-stretchable fabric, a commercially available woven polyacrylic/polyester mixed fabric with a Teflon® coating was used. The selection criteria for the fabric included water-repellency and the ability not to absorb or wick liquids even after being pierced by the needle during stitching, as well as being white to offer optimal contrast against the dyes used in the fluidic tests below.

To fabricate stretchable microfluidic textiles, sufficiently water-repellant four-way stretchable (bi-elastic) fabrics could not be identified. Instead, a commercial water-repellant product was used on a regular elastic fabric; specifically, a weft-knitted polyester/elastane mixed Scuba Crepe fabric which can be elastically stretched up to 30% in the wale direction and up to 100% in the course direction. The water-repellent used was a wax emulsion that can be dissolved in water and applied to the textile by soaking and washing (see Experimental), which gave more consistent results than spray-based alternatives. Before the treatment, the stitched microfluidic channels would leak liquid into the surrounding fabric; after the treatment, the leakage was reduced greatly (see Fig. S2†). We could determine that the treatment significantly increased the contact angle to a level close to that of the woven fabric (see Fig. 1C). However, because of the already high contact angle of the untreated knitted fabric, we do not think that hydrophobicity on the surface of the fabric is the sole factor that determines suitability for this application. When the fabric is stitched, the surface is pierced and the internal fibers could be torn, which may alter their behavior. The wicking thread is also in direct contact with the inner structure of the fabric. It is probable that the wax in the water-repellant coats the fibers in the fabric, physically blocks micro-capillaries between the filaments, and thus increases the resistance to liquid absorption. The inner structure and composition of the fabric at the fiber level are therefore likely to be just as important as the surface properties.

The size and amount of textile used in the devices we used differed greatly, but in each case, the estimated cost came down to a few euro cents based on the commercial prices. Buying in bulk for mass production would make these material costs nearly negligible compared to the overall fabrication cost, which would require electronic components such as a wearable potentiostat and biorecognition elements for biosensing.

### Stitched microfluidic channels and their behavior

Machine stitching has been the standard stitching method in the garment and soft materials industries since the Industrial Revolution, being more efficient, reproducible, and requiring less skill than the alternative of hand stitching. Conventional sewing machines typically use interlock stitching, which relies on two threads: an upper thread is fed from a spool and the lower thread from a bobbin. These threads pass on their respective side of the fabric and meet in the middle of the fabric, looping around each other at regular intervals. This is unlike hand stitching in which a single thread is alternating being the top and bottom of the fabric, generated by a motion that is hard for machines to replicate (see Fig. 1B for a schematic comparison). In machine stitching, the type, length, and width (when applicable) of the stitch, as well as the tension of the spool thread, is readily programmable with a precision that is nearly impossible to match with hand stitching. To fabricate single microfluidic channels, we used Coolmax® threads as both the spool and bobbin threads in a consumer-grade sewing machine (see Fig. 1A). In preliminary tests (see Fig. S1†), replacing either the spool or bobbin thread with a hydrophobic, non-wicking thread was attempted, but it was found that using Coolmax® thread for both gives the highest flow rate and longest flow distance, most likely because the wicking threads come in direct contact with each other and enable synergistic liquid transfer at each interlock location whereas the non-wicking thread might compress Coolmax® and block the flow.

For non-stretchable textiles, we used the woven fabric and straight interloop stitches to sew microfluidic channels. For the stretchable stitched microfluidics, we used the knitted fabric and a zigzag interlock stitch to fabricate the channels, as the zigzag stitches can stretch with the textile (see Fig. 1F), with the degree of stretching governed by the geometry of the stitching pattern.

To test the microfluidic wicking behavior of stitched channels we measured the lateral flow rate of an aqueous food dye. For the knitted fabric, we measured the flow rate and fitted the data to the Washburn equation (eqn (1)):1
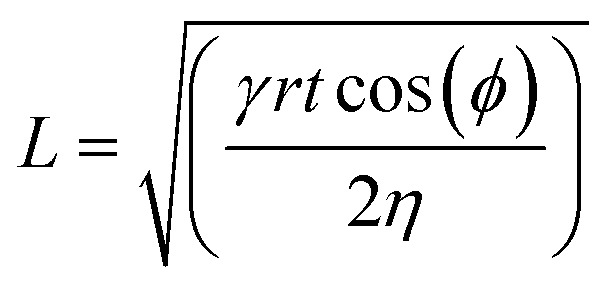
where *L* is the wicking distance, *γ* is the surface tension, *r* is the pore radius, *t* is the time, *ϕ* is the contact angle between the liquid and the yarn, and *η* is the dynamic viscosity of the liquid. Assuming that *γ*, *r*, *ϕ*, and *η* are all constant for each thread and liquid used, the distance is directly proportional to the square root of *t*, as shown in Fig. 1E. The plotted data show different wicking rates and less linear behavior compared to what we have observed in different types of unstitched Coolmax® threads in earlier work.^65^ Deviations from the linear behavior could be explained by the fact that *r* is not constant throughout the yarn as the needle and bobbin loop around each other between every stitch and squeeze each other in these spots, contracting the pores. The higher deviation in the unstretched knitted fabric could originate from liquid leaking and being absorbed by the fabric due to inefficient water-repellency (this could be remedied with a better hydrophobic coating). In the case of the zigzag stitch, the length of the thread in the stitch was calculated by multiplying the lateral displacement of the liquid by a geometrically determined ratio factor of 1.8 (calculated from the targeted width and length of the zigzag stitch and Pythagorean theorem). There will be some error in these calculations since it treats the 3D system as 2D, the thread penetrates the fabric to a certain extent, but we assumed the error this introduces for both stitch types to be negligible. Generally, we noticed that the wicking speeds were higher in the zigzag stitch than in the knitted fabric and were further enhanced by stretching. We postulate that this effect originates from a slightly higher tension in the zigzag stitch than in the straight stitch, which reduces the average pore size in the threads, thereby increasing the wicking rate. A systematic future study of stitch types, geometries, and stitch tension, as well as different types of Coolmax® threads would be needed to optimize the flow rate through changing the stitch settings.

We also tested if creasing or twisting the fabric could affect the flow behavior. Since we could not track the flow against a ruler in these arrangements, we only compared the flow distance when the flow came to a stop (see Fig. S3†). For both fabrics and corresponding stitch types, we observed that when the fabrics were twisted there were no noteworthy differences from the native states, but creasing gave rise to slightly longer flow distances. This is likely due to increased tension in the microfluidic channels when the fabric is creased.

While the performance of this type of Coolmax® yarn was deemed acceptable for the applications in this paper, we still foresee the need for a thread with a higher twist and mechanical resilience when moving to industrial applications. Care should be taken so that this change does not come at the expense of the wicking behavior.

### Advanced stitched microfluidics

**Fig. 2:**
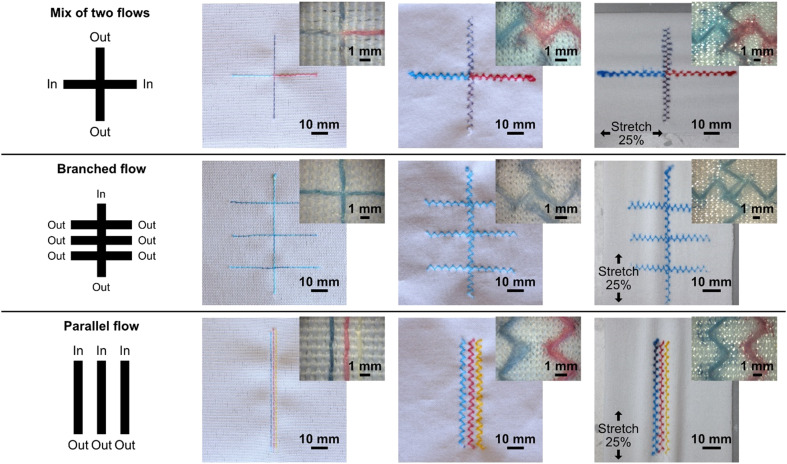
Fundamental microfluidic functions using stitched Coolmax® threads in hydrophobic fabrics. The leftmost column shows a schematic of each function. The three columns to the right show photographs of the corresponding function realized, in order: in woven, knitted, and stretched knitted fabric. The top corners show the corresponding microscope magnification of the stitches.

In this work, using our sewing machine and stitchable yarns, we could achieve a single channel width of approximately 360 μm with a minimum distance between channels of 900 μm (see Fig. 2), which is impressive given the simplicity and scale-ability of this technique. It is anticipated that more advanced equipment than ours could reduce the separation further, increasing the fabrication resolution of similar devices. Another important feature of the stitched microfluidic channels is that they allow for very efficient fluid transfer between the junction of stitched channels at the single thread scale. Fig. 2 shows single junctions with intersection areas no bigger than 360 μm × 360 μm. Using these junctions, we could achieve microfluidic fluid mixing (see Video S1†) where dyes of two different colors flow towards each other from opposing ends of a single microfluidic channel, efficiently mixing at the single junctions before being transferred to the parallel stitched yarn. We could also use several junctions to form a branched microfluidic structures and evenly split liquids (see Fig. 2). Furthermore, all these microfluidic structures could not only be integrated into the woven fabrics but also into the knitted fabric using a zigzag stitch, both at rest and when stretched up to 25%.

**Fig. 3:**
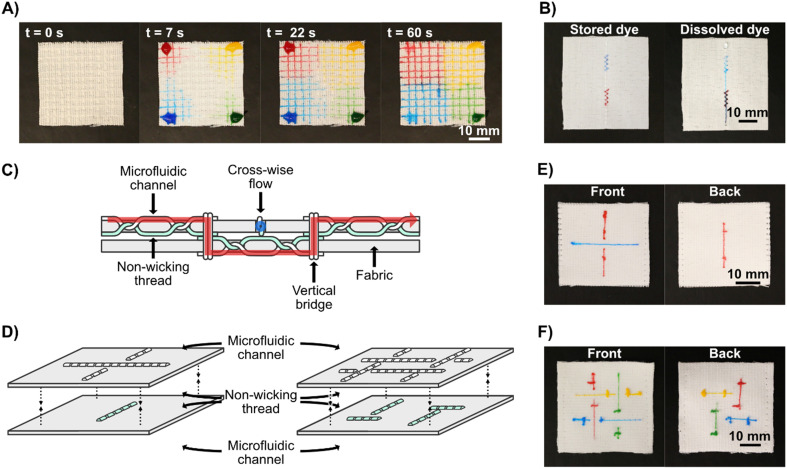
Advanced microfluidic functions in stitched textile-based microfluidic devices. A) Sequential view of liquid flow through a 4 × 4 cm stitched device with 100 microfluidic channel crossings. B) Demonstration of chemical storage in stitched Coolmax® thread. The zigzag stitches contained dried dye which was rehydrated and transported with the liquid flow after MilliQ water was added to one end of the straight stitch (top of the picture as shown). As the water passes through both storage regions, the colors of the dyes mix. C) Sideview schematic of the 3D stitched microfluidic device. The microfluidic channels of the two layers, aligned through the *z*-axis, are connected with stitches that pass through both layers vertically. Since there is no fluid path between the perpendicular flows on the top and bottom faces of the device, they do not mix. D) Oblique schematic view of the 3D stitched microfluidic device. Two layers of hydrophobic fabric are stitched with Coolmax® as the spool thread and non-wicking nylon monofilament as the bobbin thread. The faces of the fabrics with the non-wicking thread are stitched together facing each other. E) Photographs of the two faces of a complete 3D stitched textile-based microfluidic device with a single underpass crossing, showing that the flows of the two different dyes (blue and red) were kept separate. F) Photographs of the two faces of a complete 3D stitched textile-based microfluidic device with four intertangled microfluidic channels, with separated liquid flows.

The ability to integrate advanced microfluidic channels into wearable fabrics with this level of accuracy and precision allows for the upscaling of such advanced structures and implementation of many functions into relatively small textile areas. By means of an example, we show a 4 × 4 cm area having 100 junctions (see Fig. 3A) to evenly split four liquids into a matrix of different mix ratios.

For standalone, equipment-free, stitched textile systems, it may be necessary to store reagents in the device. To demonstrate the potential to store compounds in Coolmax® yarn we stitched two dense regions of zigzag stitches in the woven fabric, allowed food dye to dry in the yarn in these regions, and then stitched a straight microfluidic channel over them both. As we flowed water through the microfluidic channel, the dry food dye dissolved and flowed downstream from where it had been stored, see Fig. 3B and Video S2.†

To fabricate advanced 3D microfluidic devices, there is a need to transport fluid between several z-stacked layers and connect these layers through fluidic junctions. To achieve this, two layers of fabric were used, instead of one, each containing separate stitched microfluidic channels. In this application, we used nylon monofilament threads as the bobbin thread so that the microfluidic channel is only present on one side of the fabric. The microfluidics in the different layers could be stitched independently from each other and then stacked, wicking threads can be stitched through the two layers to act as bridges between them and non-wicking monofilaments can be used to hold the layers together without allowing fluid transport. It is important to remember that the functional threads are only exposed on one face of the fabrics, when the two layers are sewn together they can be done so to have these functional faces separated by the fabrics, to prevent fluid mixing between the faces, or with them facing each other to induce fluid transfer, as an alternative to having fluidic bridges stitched between them. We fabricated vertical fluidic channels between the layers by stitching a few stitches of Coolmax® thread as both bobbin and spool thread, crossing both fabric layers and connecting with the patterns in the fabric plane on their outer faces. Using this approach, we created simple devices with two perpendicular microfluidic channels, one which passed uninterrupted through both layers of fabric, creating an underpass crossing. After this, we created a more complex pattern of four intertangled microfluidic channels. See Fig. 3C and D for schematics of the devices, Fig. 3E and F for photographs of the respective finished devices, and Video S3.† These proof-of-concept devices show that functional three-dimensional multilayer stitched microfluidics can be fabricated. However, it is possible that high compression of the two fabrics could lead to leakage between the separated microfluidic channels and the use of microfluidic channels with just a single wicking thread should be minimized for optimal flow rates; these are points that need to be considered during the design of applied devices.

**Fig. 4:**
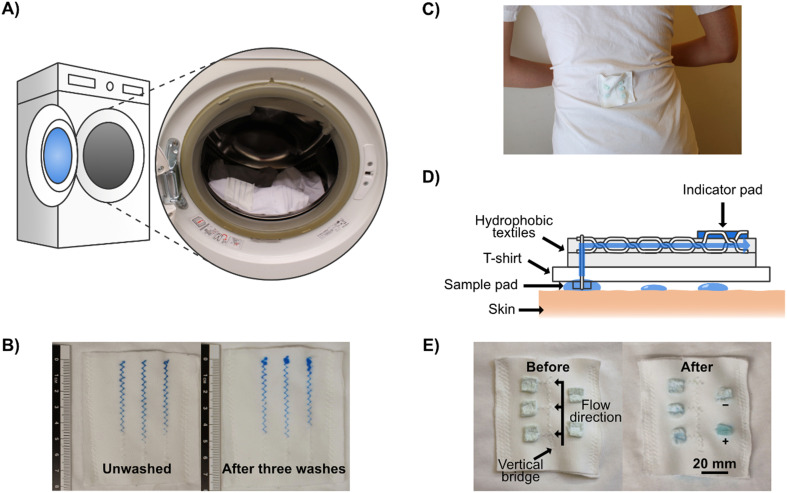
Wearable stitched textile-based microfluidic devices. A) Schematic with an embedded photograph of a T-shirt loaded into a washing machine. The T-shirt had microfluidic devices sewn onto it. B) Comparison of flow behavior in a stitched microfluidic device before the first wash and after three washes. C) Photograph demonstrating wearable stitched textile-based microfluidic devices. The microfluidic device collects and transports sweat to anhydrous copper sulfate moisture indicator pads. The microfluidic device is positioned on the lower back region of the T-shirt. D) Side-on schematic of the wearable textile-based microfluidic sweat analyzing device. E) Comparison of the wearable textile-based microfluidic sweat analysis device before and after sweat collection. The blue color in the first and second indicator pads along the microfluidic channel indicates positive collection. In the after picture, artificial sweat has been added to one of the indicator pads not connected to the microfluidic flow as a positive control.

One useful application for 3D textile devices is wearable diagnostics devices for analyzing body fluids, such as sweat. To demonstrate that sweat can be collected directly from a human body and pass through our microfluidic systems, we attached a stitched microfluidic device to a normal T-shirt, with a simple sample collection pad on the inside of the T-shirt (see Fig. S4†) and collected sweat when wearing it (see Fig. 4C for a photograph of the device when worn and Fig. 4D for a schematic illustration). Since this experiment was conducted in the Swedish winter, sweating was induced through exercise. We used cotton pads containing anhydrous copper sulfate, which turns blue in the presence of moisture, to indicate that sweat had wicked through the microfluidic channels to the cotton pads on the outside of the T-shirt. Three indicator pads were connected to the stitched microfluidic channel at different lengths from the collection pad and two out of three indicator pads turned partially blue, demonstrating that sweat had been collected from the skin and transported through the device to those indicator pads (see Fig. 4E). The viscosity, composition, and volume of sweat differ depending on the region of the body, in these tests the patch was positioned on the lower back, which for men on average has a sweat rate of around 1.8 mg cm^−2^ min^−1^.^67^

### Washing

An important feature of wearable devices is the ability to wash and re-wear them. To test this, we fabricated a simple stitched device and washed the stitched textile several times, using a standard washing machine and detergent with a washing program matching the laundry instructions of the fabric (see Fig. 4A and Experimental section for full details). We used colored dyes to assess the flow performance of the device after each washing step (see Fig. 4B). Even after three rounds of washing, we could see only a marginal decline in the wicking properties. The only issue we could observe was that the dye sometimes had problems adhering and absorbing to the end of the stitch, which could be due to residual surfactants or because the stitch started to come loose at the ends. This means that wearable stitched textile-based microfluidic devices have the potential to be reused after conventional garment cleaning.

### Integration of electrochemical sensing

**Fig. 5:**
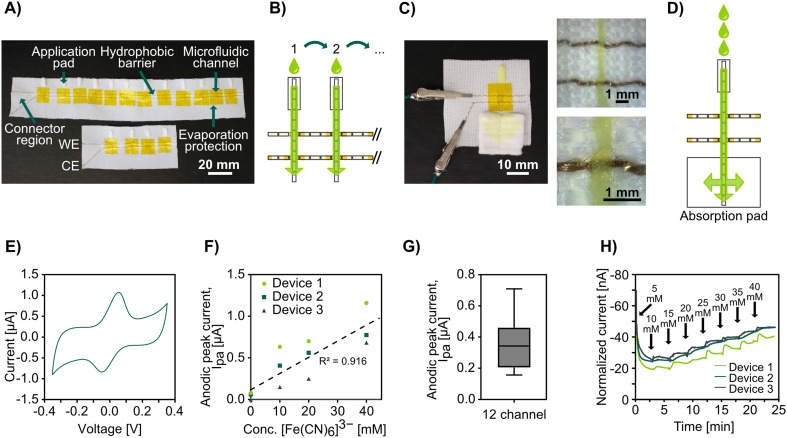
Stitched textile-based electroanalytical microfluidics. A) Photograph of stitched electroanalytical sensing devices with twelve (top) and four (bottom) microfluidic channels with components labeled in the images. B) Schematic of the flow in microfluidic channels across stitched electrodes in the electrofluidic device showing the microfluidic Coolmax® in white, the Au-coated threads in gold, and the fluid flow in green. C) Photograph of a stitched electroanalytical device capable of continuous monitoring during flow (top side of the device shown in the picture) with embedded microscope images of the intersection between the microfluidic channel and the electrodes. D) Schematic of flow in the electrofluidic device capable of continuous monitoring during flow, showing the microfluidic Coolmax® in white, the Au-coated threads in gold, and the fluid flow in green. E) An example cyclic voltammogram of 40 mM ferricyanide in the stitched electroanalytical device at a scan rate of 30 mV s^−1^. F) Plot of anodic peak current *versus* analyte concentration, *n* = 3. G) Box plot of anodic peak current from twelve microfluidic channels on the same stitched electroanalytical device. A ferricyanide concentration of 30 mM was used in all channels. H) Normalized chronoamperometric data under the continuous flow solutions with increasing ferricyanide concentrations in three stitched electroanalytical devices.

To demonstrate the possibility of combining stitched microfluidics with electrochemical detection of analytes, we integrated gold-coated threads as stitched electrodes. We have previously shown that these threads can be used as biosensors by attaching biomolecules to them using Au-thiolate self-assembled monolayer chemistry (without the requirement for any advanced treatment) and used as standalone thread electrodes or in wearable textiles.^65,66,68,69^ Here we stitched the gold-coated threads in parallel stitches, using the Au-coated thread as both the bobbin and spool thread. By sewing Coolmax® microfluidic channels perpendicular to the gold-coated yarn we could create electrochemically active micro junctions (see Fig. 5B). We used rectangular pieces of non-woven cotton pads at one end of each microfluidic channel to act as sample application pads, similar to sample pads in lateral flow assays.

The designs with two parallel gold-coated yarns formed a two-electrode system, and we coated the stitches by drop casting with a hydrophobic polymer on either side of the junctions to create a barrier that prevents the solutions from wicking through the conductive threads to the connectors and shorting the circuit. Pieces of tape were used to cover the sensing junctions on both faces of the fabric to prevent evaporation. See Fig. 5A for photographs of the devices and Fig. 5B for a schematic. We connected the gold-coated stitched electrodes directly to a potentiostat, added ferricyanide solutions to the sample application pads, waited around 10 seconds to allow the entire microfluidic channel and electrodes to wet, and then performed cyclic voltammetry (CV) to analyze the electrochemical response of the conductive threads at the wetting junctions. Afterward, we removed the tape covering the sensing region and allowed the electrodes to dry completely before applying a sample to the next sample pad. The obtained voltammograms (see Fig. 5E) showed clear and distinct redox peaks, demonstrating that we could perform electrochemical detection on this simple platform with only a micrometer-scale overlap between the stitched microfluidic channels and electrodes. During early experiments without the use of tape, the results showed a constant decrease in peak height for every cycle (see Fig. S6†), which we speculated to be due to evaporation (Coolmax® is designed to enable fast evaporation) and drying of the electrode, and this issue was mended by using tape as a seal. While evaporation would also lead to condensation of the analyte, we believe that the loss of the effective electrode surface area due to had a greater impact on the monitored currents, which would explain the observed trend. Although not attempted in this study, we believe it could be possible to utilize this innate evaporation to concentrate samples for increased sensitivity in future applications.

Using this above-mentioned approach, we fabricated devices with four stitched microfluidic channels (each crossing the same two conductive threads, forming an array of four parallel working electrodes), measured four solutions with varying concentrations of ferricyanide, and were able to show the expected linear current response (see Fig. 5F).

Further, to show the potential for scaling these electroanalytical textile devices, we fabricated devices with twelve stitched microfluidic channels and performed CV measurements as above with the same concentration of ferricyanide in the applied solution. Fig. 5G shows the average oxidation peak currents from the voltammograms obtained for all twelve channels. While the data has a high standard deviation, this was attributed to the handmade nature of these devices. The peak current is directly influenced by the electrode surface area per the Randles–Sevcik equation. Variations in the electrode surface could have arisen from the manual coating or varying thickness and density throughout the threads, which is affected by the tension as well as inconsistency during the manufacturing of the thread. We believe the issue with high standard deviation could be overcome with further optimization of device fabrication, including using more standardized masking and coating techniques when defining the sensing region.

For many analytes, it is important to monitor how their concentration changes over time. Therefore, we developed a microfluidic device capable of continuous monitoring. To this end, we stitched a single microfluidic channel with two parallel gold-coated threads as working and counter electrodes that had a smaller sample application pad (fluid inlet) and a much larger absorption pad (fluid outlet), see Fig. 5C for a photograph and Fig. 5D for a schematic. The absorption pad continuously drove the flow even after the Coolmax® yarn had been completely wetted, as in lateral flow assays. We pipetted ferricyanide solutions of increasing concentrations periodically to the inlet pads and monitored the step-wise change in concentration with chronoamperometry. These devices had a high drift (see Fig. S7† for raw data). However, after normalizing the data against the drift the amperograms (see Fig. 5H) showed the expected ladder shape with changes in redox agent concentration. It should, however, be noted that when we attempted to go from high to low concentration, the current response did not go down proportionally (see Fig. S8†). We believe this was caused by an accumulation of the analyte at the electrode surface and it is possible that this could be solved by surface treatment of the electrode or by making the microfluidic channel cover a larger area of the electrode surface for better wash effect.

While the CVs showed a reduction peak at around −0.05 V, we used a more negative potential of −0.15 V for our chronoamperometry experiments because we found this resulted in higher currents and more discrete plateau levels. It should be noted that shifts in the reduction potential are to be expected due to the lack of a standard reference electrode.

It is encouraging that we have been able to establish the principle of real-time, continuous quantifiable monitoring of analytes under flow in a completely wearable and reusable system. For ease of fabrication, the fabric substrate of all electrofluidic devices was the non-stretching woven fabric. We are confident, however, that the patterns used could be translated to zig-zag stitches and applied on the stretchable knitted fabric. In our previous work, we have shown that electrochemical measurements on stitched gold thread electrodes are not significantly affected by stretching.^68^ It is easy to see how this proof-of-principle stitched device can be further developed to create the next generation of wearable textile-based analytical devices.

It is worth noting that all of these advanced stitched textile-based microfluidic devices were stitched by researchers with no prior professional sewing experience. With just minimal training we were able to fabricate structures and patterns that would be very challenging and time-consuming to do by hand. The lock-stitch method used in this project and the specific structures received from its use are only practically possible with a commercial sewing machine. Trained professionals or the automatic sewing machines found in industrial settings are likely to be much more efficient and achieve more consistent fabrication results.

## Conclusion

We have shown that it is possible to fabricate advanced textile-based microfluidics by machine-stitching Coolmax® threads into hydrophobic, water-repellent fabrics. We have demonstrated that it is possible to perform several microfluidic functions including mixing, splitting, and multilayer three-dimensional microfluidics. The stitched microfluidic channels could be as small as 360 μm in width and by overlapping stitches, we could form junctions that enabled highly efficient liquid transfer. Importantly, we could easily form robust microfluidic systems capable of stretching up to 25% using zig-zag stitch in stretchable textiles. We also integrated stitches microfluidics into wearable clothes for collecting and transporting sweat from real human skin, for optical readout. Furthermore, we have demonstrated the possibility of integrating electrochemical detection of analytes by stitching electrodes of gold plasma-coated polyester threads crossing the stitched microfluidic channels. These junctions, which have only micrometer-scale overlaps, acted as electrodes and could detect analytes in both stationary liquids and mobile flow.

Our stitched textile-based microfluidics relies on a top-down fabrication method based on machine stitching, which is inherently scalable and allows advanced microfluidic devices to be easily realized at laboratory scale as well as scaled up industrially. It therefore has great potential to be utilized for wearable microfluidic analytical platforms that are cheap and reusable. Future work should focus on further characterizing the microfluidic flows in these systems to facilitate optimization of the fluidic functions and open up new methods of sample manipulation, as well as moving toward relevant bio-sensing applications. Challenges that remain include: i) the development of robust interconnects between the conductive textile parts and external electronic components (*e.g.* a wearable potentiostat), and ii) the integration of functionalized gold-coated threads for biosensing applications (*e.g.* through self-assembled monolayers with biorecognition elements).

## Data availability

The data is available on servers at KTH dedicated as data repository.

## Author contributions

Martin Hanze: conceptualization, methodology, investigation, formal analysis, visualization, writing – original draft. Andrew Piper: conceptualization, writing – review & editing. Mahiar M. Hamedi: conceptualization, writing – review & editing, supervision, funding acquisition, project administration.

## Conflicts of interest

There are no conflicts to declare.

## Supplementary Material

LC-025-D4LC00697F-s001

LC-025-D4LC00697F-s002
