# Supplementary material for: Stitched textile-based microfluidics for wearable devices
Source: Lab Chip. 2024 Nov 18;25(1):28–40. doi: 10.1039/d4lc00697f (PMC11599943; doi:10.1039/d4lc00697f)
Supplement: LC-025-D4LC00697F-s002 [file LC-025-D4LC00697F-s002.pdf]

Supplementary Information (SI) for Lab on a Chip.
This journal is © The Royal Society of Chemistry 2024

SUPPORTING INFORMATION

Stitched textile-based microfluidics for wearable devices

Martin Hanze[1], Andrew Piper[1,2*], Mahiar M. Hamedi[1*]

[1] Department of Fibre and Polymer Technology, School of Engineering Sciences in Chemistry, Biotechnology and Health, KTH Royal Institute of Technology, Teknikringen 56-58, SE-100 44, Stockholm, Sweden
[2] Institut Català de Nanociència i Nanotecnologia (ICN2), CSIC and The Barcelona Institute of Science and Technology (BIST), Campus UAB, Bellaterra, 08193 Barcelona, Spain

[*] Corresponding authors: Andrew Piper (andrew.piper@icn2.cat), Mahiar M. Hamedi (mahiar@kth.se).

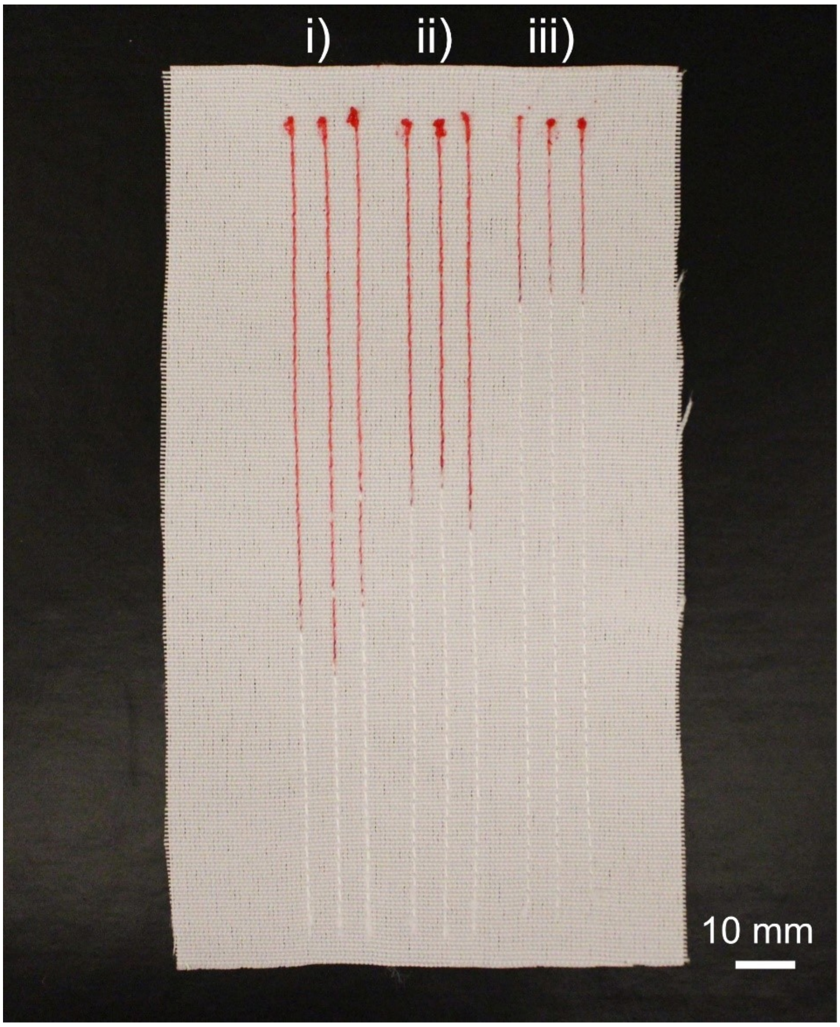

Figure S1: Comparison of flow distance in stitched microfluidic channels in the woven fabric using straight stitches with i) Coolmax® as both spool and bobbin thread, ii) Coolmax® as spool thread and non-wicking nylon thread as bobbin thread, and iii) nylon thread as spool thread and Coolmax® as bobbin thread (the latter stitched with the fabric upside down so that the microfluidic channels are visible in all three cases from the same side).

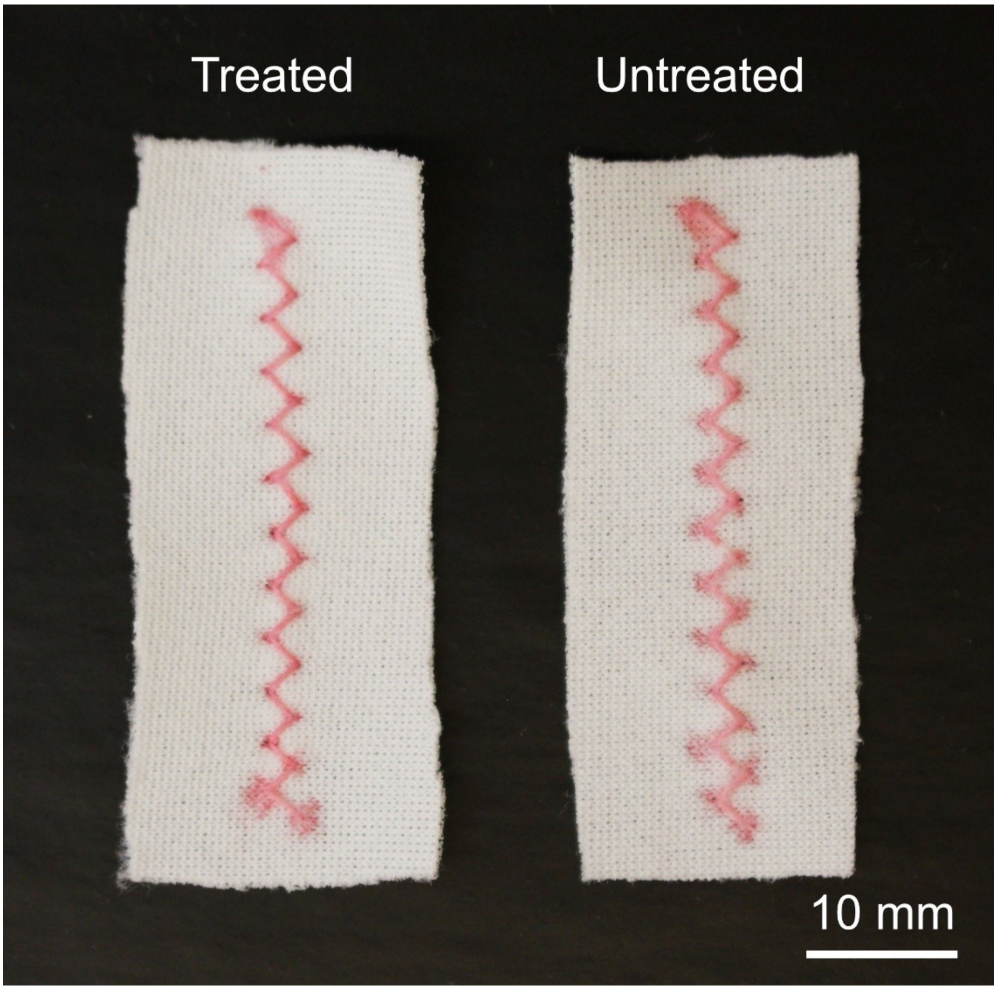

Figure S2: Comparison of water-repellant treated knitted fabric and untreated fabric.

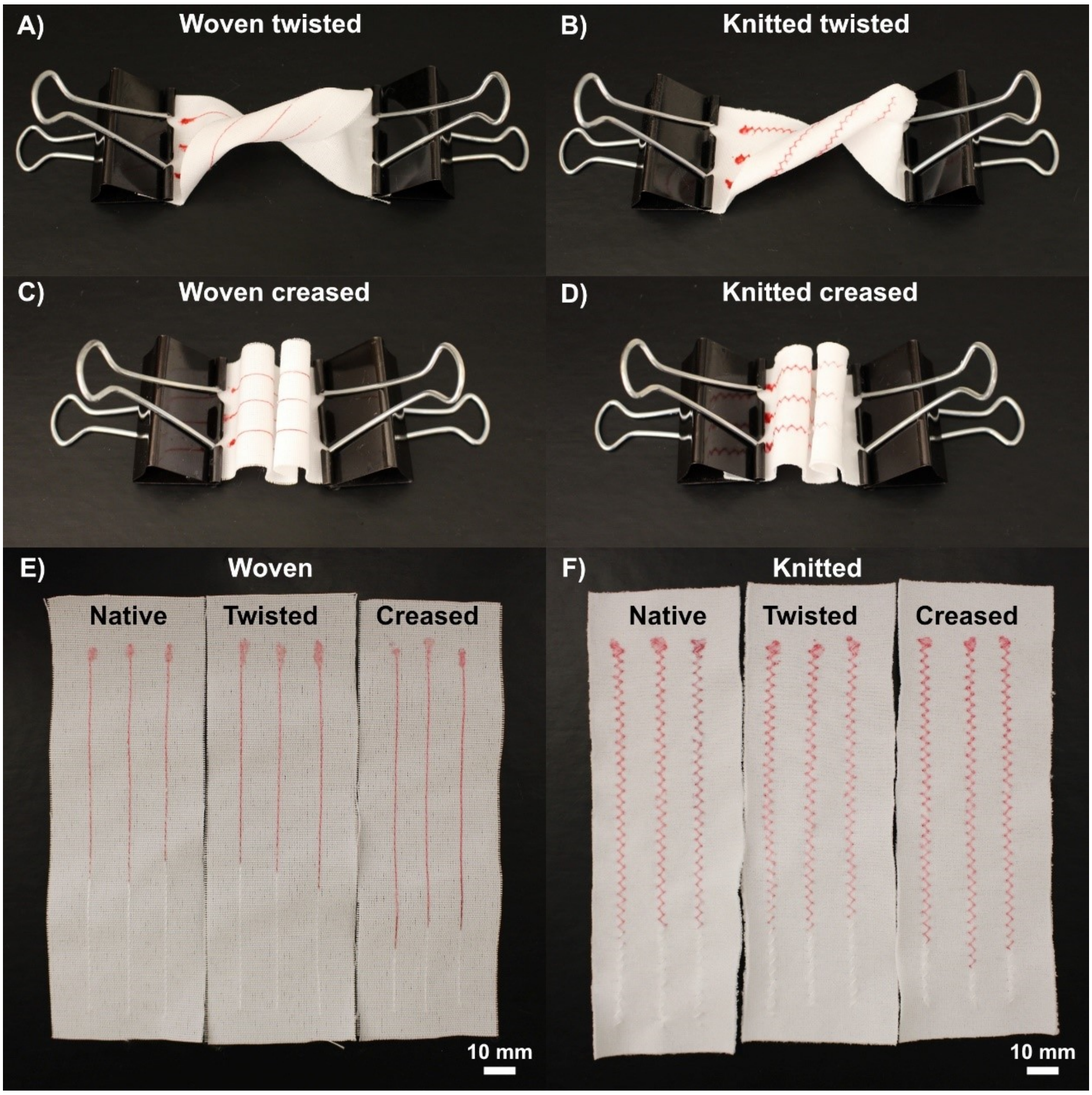

Figure S3: Comparison of flow distance in stitched microfluidic channels when the fabric is twisted or creased compared to the native state. **A)** and **B)** show how the woven and knitted fabrics are twisted 360 degrees. **C)** and **D)** show how the woven and knitted fabrics are creased with three creases. **E)** and **F)** show the comparison of the flow distance between the twisted and creased fabrics and the native state for the woven and knitted fabrics, respectively.

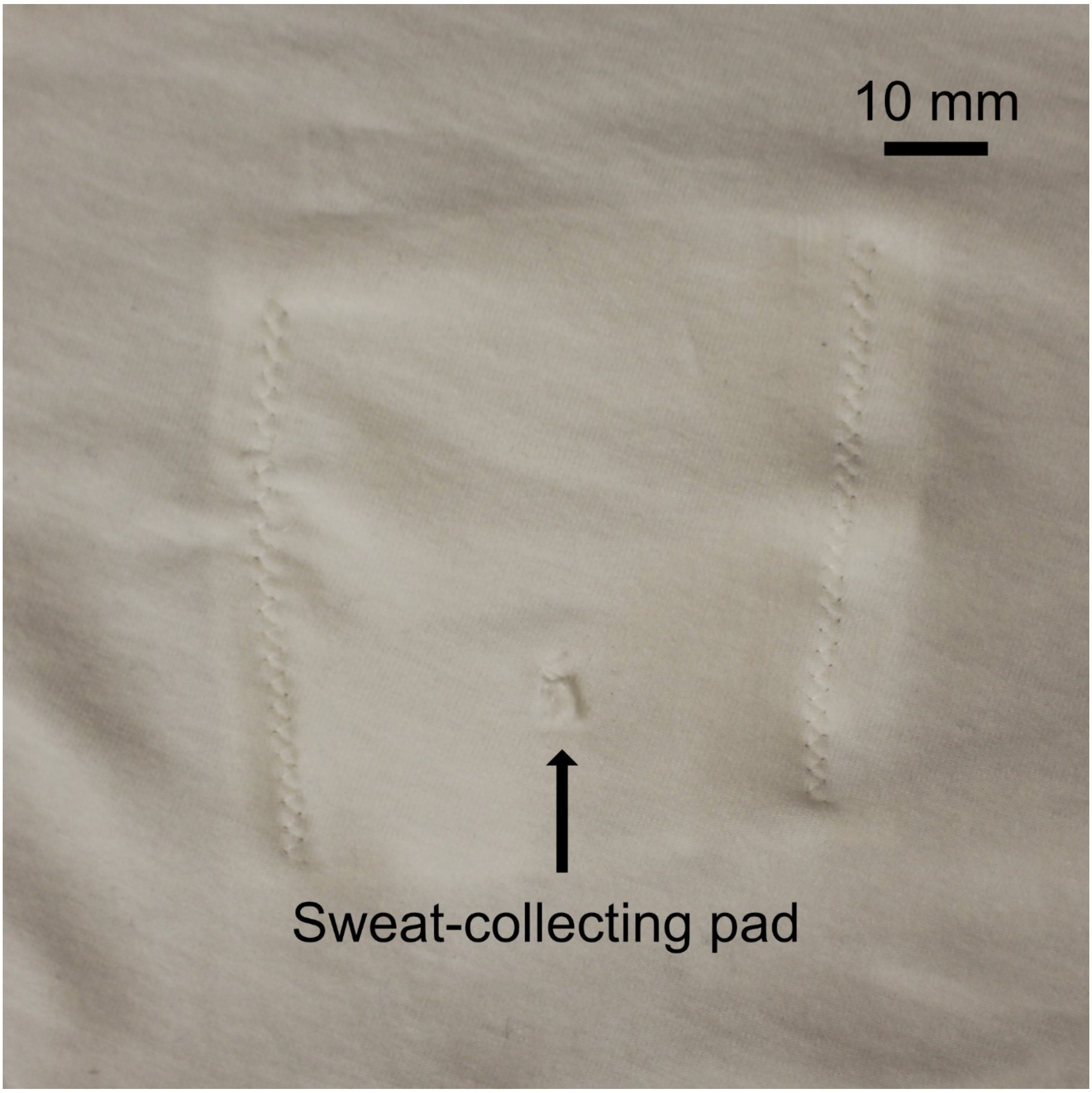

Figure S4: Photograph from inside the T-shirt with the sweat-indicating stitched microfluidic device attached to it on the outside (not visible), before use. Visible are the stitches with non-wicking nylon thread that connects the device to the T-shirt's fabric and the sweat-collecting pad made from a non-woven cotton pad that is the only part of the device on the inside of the T-shirt and touches the skin of the wearer, isolating the user from any reactants.

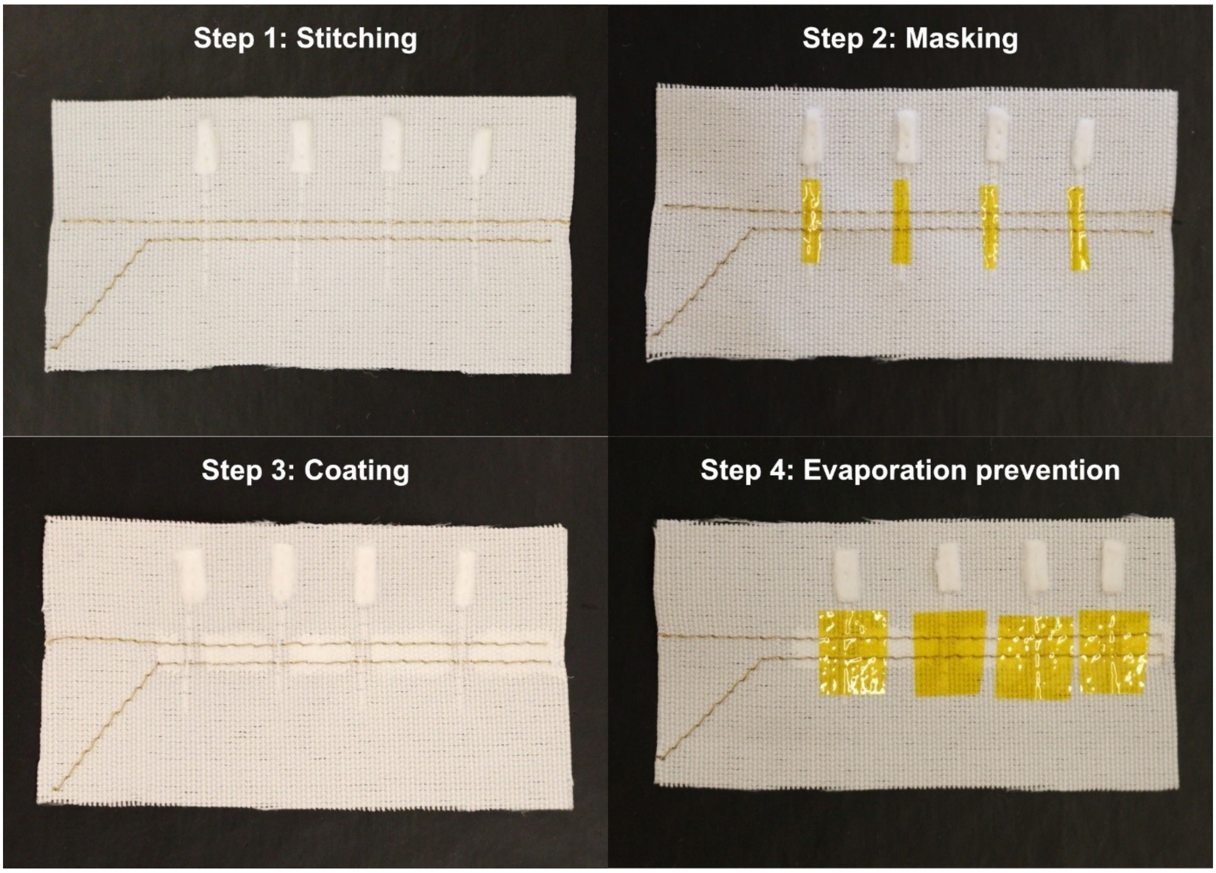

Figure S5: Step-by step documentation of the fabrication of stitched electroanalytical devices. In the first step, the gold yarn is first stitched as electrodes. After that, the microfluidic channels are stitched perpendicular to the electrodes, starting from the cotton sample application pads which are attached to the fabric substrate through the stitching. The microfluidic channels need to be stitched after the electrodes to get a proper wetting of the electrodes. In the second step, the sensing region is masked with pieces of tape. In the third step, the electrodes are coated with a hydrophobic polymer (nail varnish), the mask is removed, and the coating is allowed to dry completely. In the final step, the sensing regions are covered with tape to prevent evaporation. Note: Masking, coating, and evaporation prevention are done on both sides of the devices (not shown).

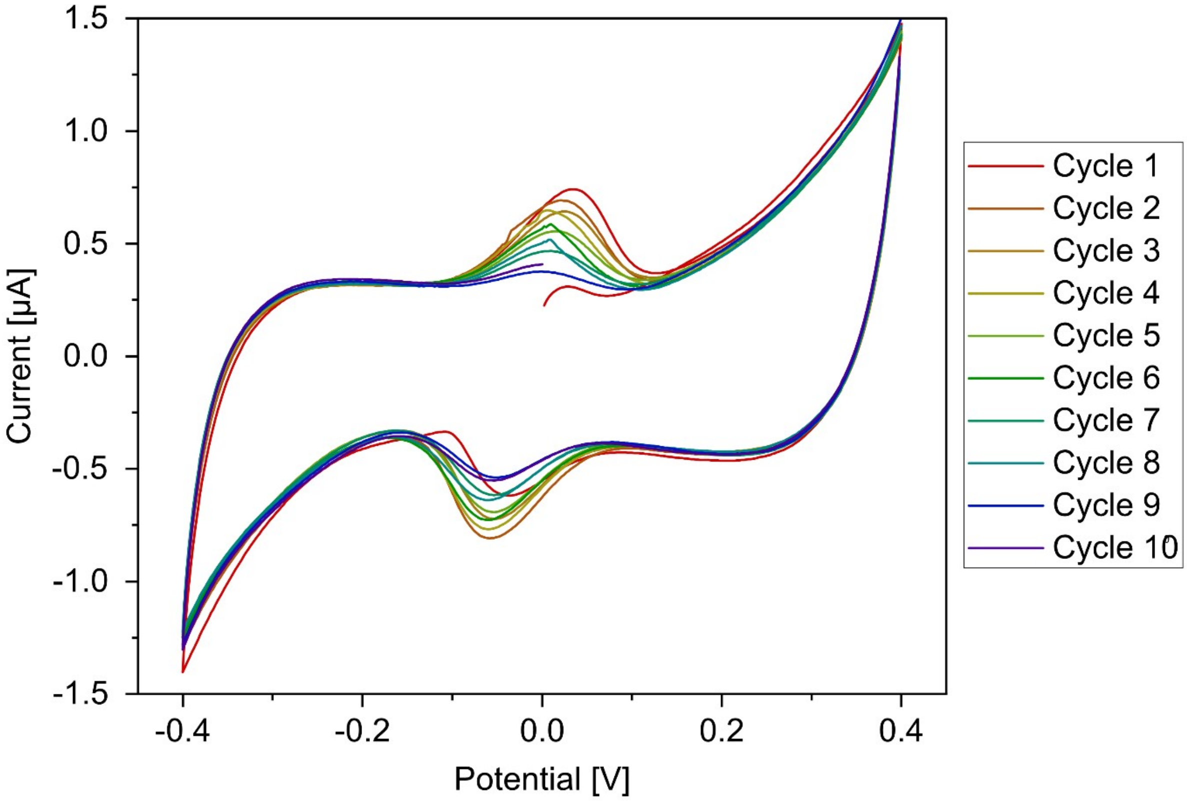

Figure S6: Voltammogram of an early stitched electroanalytical device without tape seals covering the sensing region. 10 cycles were performed on 10 mM ferricyanide solutions at a scan rate of 10 mV/s.

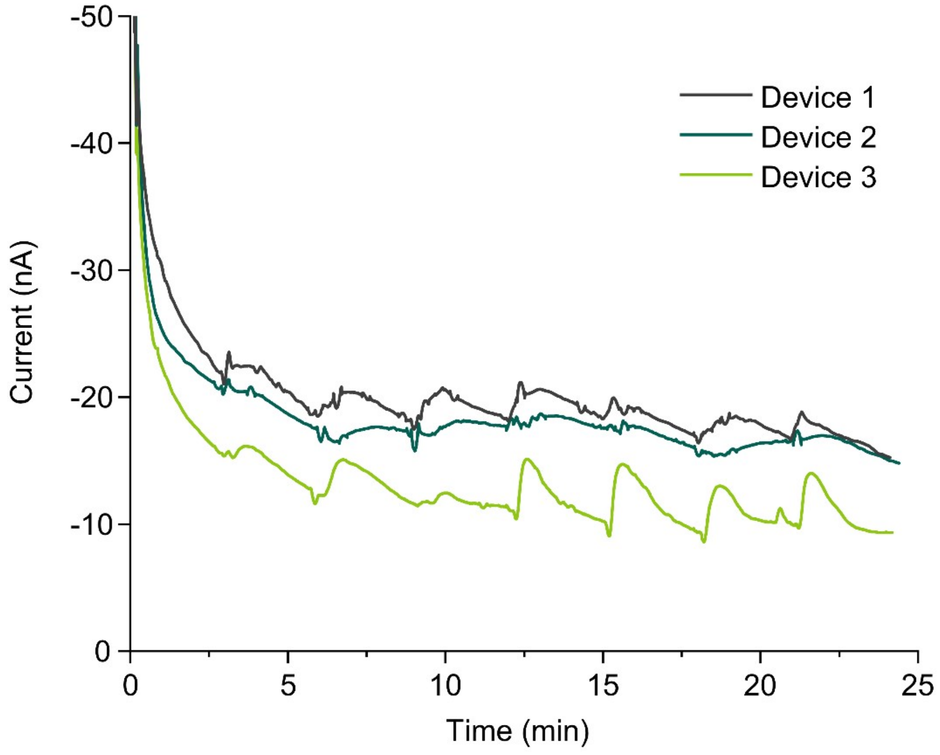

Figure S7: Chronoamperometry data before normalization. The data is collected under the continuous flow of ferricyanide solutions with increasing concentrations every 3 minutes in three stitched electroanalytical devices.

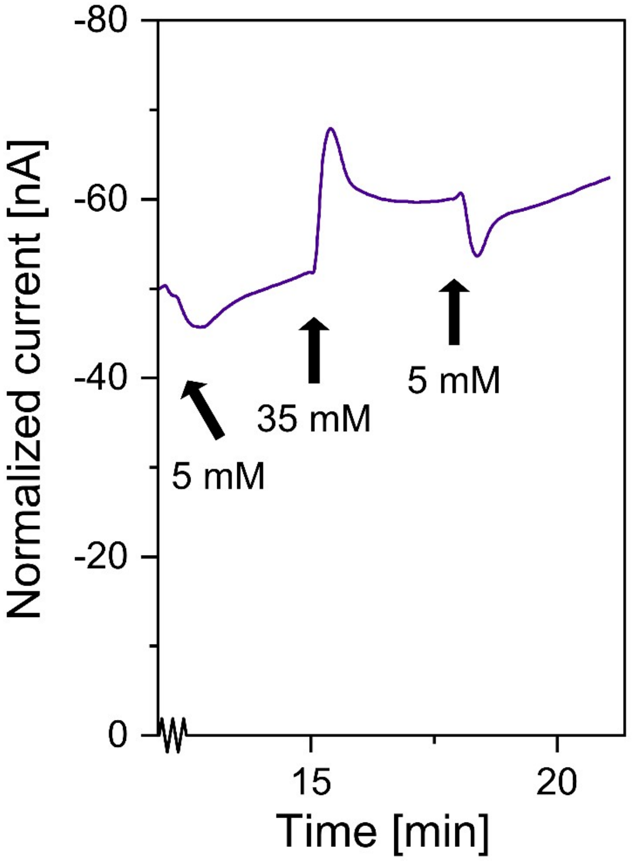

Figure S8: Chronoamperometric data that show the behavior in a stitched continuous flow device when going from a high to a low concentration. As can be seen, the change in current when going from 35 mM to 5 mM is less than going from 5 mM to 35 mM. The data has been normalized in the same manner as in Figure 5H and truncated for clarity.

Table S1: Specification of all the yarns and threads used.

Yarn name	Type	Yarn number	Filament no.	Diameter (µm)	Supplier
Coolmax® Ecomade	Polyester yarn	dtex 78/48/2	156	Unspecified	Dúctel, s.a.
Gold plasma coated yarn Polyester FDY, high bright (1.1 mg/m Au)	Plasma-coated polyester yarn	dtex 125/f36/2	72	246 ± 20	Swicofil AG
Transparent nylon thread	Monofilament nylon thread	dtex 356	1	120	Selfmade Sverige AB

Table S2: Specifications of all the fabrics used.

Fabric name	Materials	Type	Weight (g/m^2)	Supplier
Outdoor Fabric Teflon Plain – white	60% Dralon® (polyacrylic), 40% polyester	Plain weave	180.00	fabfab GmbH
Lightweight Crepe Scuba – white	94% polyester, 6% elastane	Warp knitted	220.00	fabfab GmbH

Appended videos

Video S1: Mix of two flows of aqueous solutions in a cross of Coolmax® microfluidic channels.

Video S2: Dissolving stored compounds in a microfluidic channel.

Video S3: Flows in a stitched three-dimensional microfluidic device.
